# Case Report: Retroperitoneal dedifferentiated solitary fibrous tumor with unexpected Pax-2 expression, mimicking high grade clear cell renal cell carcinoma

**DOI:** 10.3389/fonc.2025.1569160

**Published:** 2025-05-13

**Authors:** Jia Wei, Chen-chen Yao, Yue-Fen Zou, Yong-Zhong Wei, Qin-he Fan, Qi-xing Gong

**Affiliations:** ^1^ Department of Pathology, The First Affiliated Hospital of Nanjing Medical University, Nanjing, China; ^2^ Department of Pathology, Women’s and Children’s Hospital Affiliated to Xiamen University (Xiamen Maternal and Child Health Care Hospital), Xiamen, China; ^3^ Department of Radiology, The First Affiliated Hospital of Nanjing Medical University, Nanjing, China; ^4^ Department of Orthopedics, The First Affiliated Hospital of Nanjing Medical University, Nanjing, China

**Keywords:** solitary fibrous tumor, dedifferentiated, STAT6, Pax-2, P53, next generation sequencing

## Abstract

Here, we report a case of retroperitoneal solitary fibrous tumor (SFT) in a 69-year-old Chinese woman. The patient had experienced lumbodorsal pain for more than two years. A computed tomography (CT) scan showed a mass adjacent to the right kidney, invading the surrounding soft tissues, measuring about 9.3 × 8.4 cm. An incision biopsy was performed. Microscopically, the tumor was composed of sheets of epithelioid cells with round to ovoid nuclei, and abundant clear cytoplasm. The cells showed severe nuclear atypia and brisk mitosis, with thin-walled branched blood vessels set against a myxoid to collagenous background. By immunohistochemistry, the tumor cells exhibited diffuse and strong expression of CK-pan, Pax-2, P53, INI-1 and H3K27me3. Staining for CD34, S100, SOX10, TLE1, WT-1, and CK5/6 was negative. STAT6 staining was weak and indistinct. Furthermore, next generation sequencing (NGS) disclosed a rare *NAB2-STAT6* (N5::S16) gene fusion, accompanied by a C141G missense mutation of TP53 gene. Consequently, a diagnosis of dedifferentiated SFT (DSFT) was determined, rather than high-grade clear cell renal cell carcinoma. The case demonstrated that DSFTs are prone to be misdiagnosed, particularly in atypical locations with abnormal morphology and immunophenotypes. In such circumstances, a comprehensive evaluation of clinical, pathological, and imaging studies is essential, and molecular examinations can provide valuable diagnostic support.

## Introduction

Solitary fibrous tumor (SFT) is a mesenchymal tumor consisting of spindle-shaped to ovoid cells arranged in a “patternless pattern” with a collagenous background and “staghorn” vessels as morphologic features. At the molecular level, it harbors the characteristic NAB2-STAT6 gene fusion. Dedifferentiated solitary fibrous tumor (DSFT) is an extremely rare subtype of SFT that presents as a high-grade sarcoma, with or without heterogenous components (1). DSFT can occur across a wide range of ages and anatomical sites, most commonly in middle-aged adults, and is prevalent in the retroperitoneum and deep soft tissues (2). Morphologically, DSFT lacks classical SFT pattern, exhibiting high-grade sarcomatoid features, with tumor cells diffusely or disorderly arranged. Immunohistochemically, tumor cells often lose the expression of CD34 and STAT6, which makes the diagnosis more difficult (2). Here, we report a case of retroperitoneal DSFT with high-grade epithelioid morphology and an abnormal immunophenotype that mimics high-grade renal clear cell carcinoma.

## Case presentation

**Figure 1:**
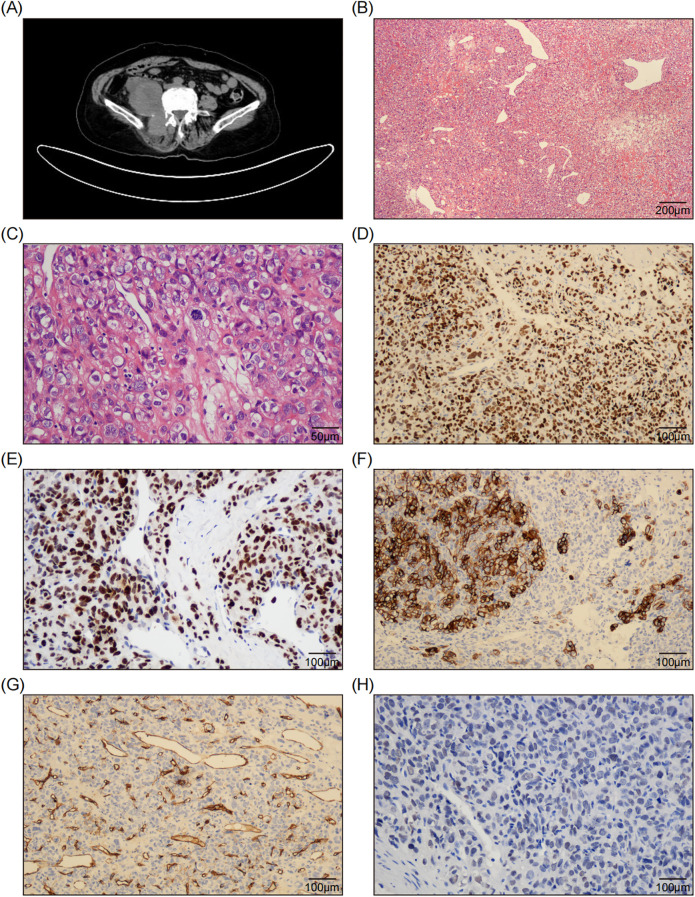
Radiological and Pathological Features of the Tumor. **(A)** The CT scan shows a right retroperitoneal mass adjacent to the psoas major and erector spinae muscles, and the right kidney was compressed and displaced. **(B)** The H&E staining image demonstrates the presence of sheets of epithelioid cells interspersed with abundant thin-walled blood vessels and focal fibromyxoid stroma. (100x; Scale bar: 200 μm.) **(C)** A representative high-magnification microscopic image reveals tumor cells characterized by round to ovoid nuclei, abundant clear cytoplasm, well-defined cell membranes, large and bizarre nuclei, coarse and vacuolated nuclear chromatin, sporadic nucleoli, pronounced atypical features, and active mitotic activity. (400x; Scale bar: 50 μm.) **(D, E)** The immunohistochemical staining images reveal diffuse and strong expression of Pax-2 **(D)** and P53 **(E)** in tumor cells. (200x; Scale bar: 100 μm.) **(F)** The immunohistochemical staining image reveals focal positive expression of CK7 in tumor cells. (200x; Scale bar: 100 μm.) **(G)** The immunohistochemical staining image reveals CD34 negativity in tumor cells, while indicating positive expression in the walls of blood vessels. (200x; Scale bar: 100 μm.) **(H)** The immunohistochemical staining image reveals weak and indistinct expression of SATA6 in tumor cells. (200x; Scale bar: 100 μm.).

A 69-year-old female patient had suffered lumbodorsal pain for more than two years, along with right thigh pain and occasional numbness. No treatment had been taken other than physical therapy. In the past month, the patient began to experience a worsening pain. A CT scan revealed a cystic-solid mass measuring approximately 9.3 × 8.4 cm in size (**Figure 1A**), located adjacent to the right kidney, invading the right psoas major and erector spinae muscles, extending to the spinal canal of L4-L5 level thorough the right intervertebral foramen. This was suspected to be a malignant peripheral nerve sheath tumor. The patient was scheduled to undergo a lumpectomy. However, during the surgery, it was observed that the tumor had invaded the surrounding tissues, making surgical removal challenging. An incision biopsy was performed.

Microscopically, the tumor was composed of sheets of epithelioid cells interspersed with abundant thin-walled blood vessels (**Figure 1B**). At higher magnification, the tumor cells exhibited round to ovoid nuclei and abundant eosinophilic to clear cytoplasm surrounded by distinct cell membranes, which prompted an initial consideration of renal clear cell carcinoma, particularly given the tumor’s pararenal location. The nuclei were large and bizarre, with rough and vacuolated nuclear chromatin, and occasionally discernible nucleoli. The cells showed severe atypia and brisk mitosis (22/10HPF) (**Figure 1C**). Tumor necrosis was absent in the biopsy tissue. Some areas of fibromyxoid stroma were observed, as well as focal collagen bands. Given the high-grade epithelioid malignant morphology, several differential diagnoses were considered, including epithelioid malignant peripheral nerve sheath tumor (MPNST), proximal epithelioid sarcoma, pleomorphic undifferentiated sarcoma, and poorly differentiated carcinoma.

Immunohistochemically, the tumor cells exhibited diffuse and strong expression of CK-pan, Pax-2 (**Figure 1D**), P53 (**Figure 1E**) and INI-1, along with focal positive expression of CK7 (**Figure 1F**), EMA, CA9, and CD10. Staining for S100, SOX10, CD34 (**Figure 1G**), TLE1, CD99, NKX2.2, WT-1, Pax-8, and CK5/6 was negative. H3K27me3 staining was intact. The Ki-67 labeling index was approximately 50%. This perplexing immunophenotype led us to misdiagnose the condition as high-grade renal clear cell carcinoma.

**Figure 2:**
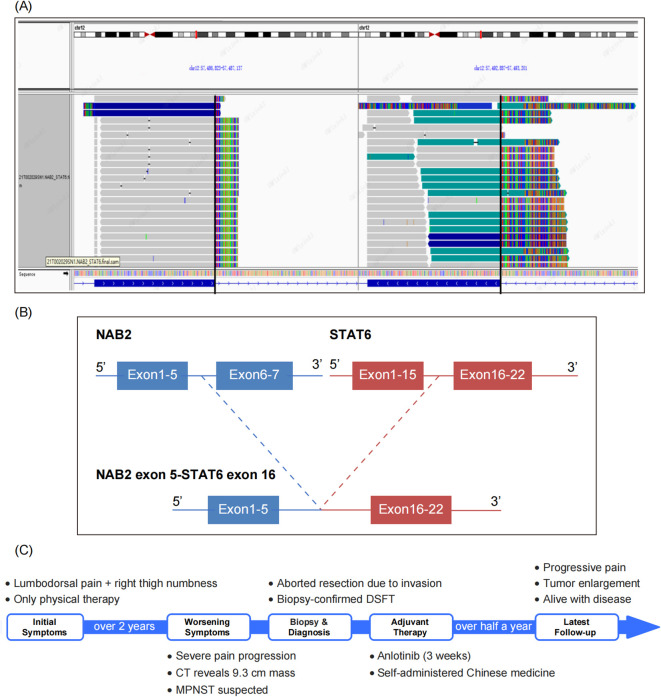
NGS Identifies a Rare *NAB2-STAT6* (N5::S16) Gene Fusion. **(A)** The Integrative Genomics Viewer (IGV) plot illustrates a rare *NAB2-STAT6*(N5::S16) gene fusion detected by DNA-based and RNA-based next generation sequencing (NGS). **(B)** The gene structure diagram illustrates the *NAB2-STAT6* fusion event, with detailed breakpoints where the N5 exon of *NAB2* is fused to the S16 exon of *STAT6*, which speculated to result in a functional oncoprotein. **(C)** The visualized timeline shows the progression of the patient’s condition.

However, clinicians and radiologists strongly believed that it was a soft tissue sarcoma rather than of epithelial origin. Upon reviewing the slides again, the “staghorn” blood vessels and focal collagen bundles captured our attention. We considered the possibility of a malignant SFT and performed a STAT6 test using immunohistochemistry and next-generation sequencing (NGS). The STAT6 staining was weak and indistinct (**Figure 1H**). Meanwhile, DNA-based and RNA-based NGS testing revealed a rare *NAB2-STAT6* (N5::S16) gene fusion (**Figures 2A, B**), along with a C141G missense mutation of *TP53* gene. Consequently, a diagnosis of dedifferentiated SFT (DSFT) was established.

Postoperatively, the patient received anlotinib as adjuvant therapy for 3 weeks. However, the treatment had limited effectiveness, and was accompanied by severe side effects. Subsequently, the patient was discharged, and began self-administering Chinese medicine. Half a year later, the patient was alive with tumor, while the severity of pain was increasing, and a CT scan revealed significant enlargement of the tumor. The visualized timeline is shown in **Figure 2C**.

## Discussion

DSFTs display a biphasic pattern composed of a conventional SFT area and a dedifferentiated morphology component with increased cellular atypia and mitotic activity synchronically or heterochronously (2). The dedifferentiated area lacks the typical SFT morphology and shows high-grade tumor cells with solid sheet-like pattern or disordered arrangement (3). The tumor cells exhibit various morphologies, including pleomorphic, spindle, epithelioid, and small round cells (4). These components can be observed individually or in combination. Sometimes, the dedifferentiated area is characterized by a heterogeneous component, which may include rhabdomyosarcoma, osteosarcoma, chondrosarcoma, *etc (* 2, 5). The transition between the dedifferentiated area and the classic SFT area is often abrupt (4), and sometimes the dedifferentiated area may gradually transition or become embedded in the classic SFT (6). DSFTs commonly present with necrosis, cystic degeneration, significant nuclear heterogeneity, and brisk mitoses (4). In our case, the classic SFT morphology was not observed probably owing to the biopsy tissue. However, the long term medical history suggested that the high grade malignant morphology might be transformed from benign or low grade tumors.

Immunohistochemically, STAT6, as a specific diagnostic marker for SFT, shows diffuse nuclear-positive expression in classical SFT, whereas it may lack typical expression in DSFT, which is negative or weakened in 82% of DSFT cases, showing patchy or focal positivity (7). In this case, STAT6 staining was weak and patchy, which was consistent with DSFT, but the weak staining of STAT6 was insufficient to the diagnosis. In fact, STAT6 may show patchy or focal positive expression in other soft tissue tumors, such as dedifferentiated liposarcoma, clear cell sarcoma, and malignant peripheral nerve sheath meningiomas (8), *etc.* Thus, there is a risk of diagnosing SFT when STAT6 is atypically expressed. Moreover, SFTs with absent or weakened STAT6 immunoexpression often show concomitant CD34 expression deficiency, and 50% of DSFTs, as reported in the literature, do not express CD34 in their dedifferentiated regions (7), making the diagnosis and differential diagnosis of DSFT more difficult. In addition to the abnormal immunoexpression of CD34 and STAT6, this case also showed positive expression of cytokeratin (CK) and Pax-2. These findings, combined with the particular site and morphological features, initially resulted in a misdiagnosis of renal cell carcinoma owing to the biopsy samples and insufficient morphological observation. Afterwards, by consulting the literature, we found that in a series of studies of retroperitoneal SFTs, 43% of the tumors exhibited positive expression of Pax-8, and 26.7% of the cases showed diffuse nuclear positivity (9). McDaniel et al. (10) performed immunohistochemical analyses of Pax-2 and Pax-8 in 41 SFTs occurring in different sites. The study revealed that the expression rates of Pax-2 and Pax-8 were 12.2% and 26.8%, respectively. However, no significant correlation was found between their expression levels and the age, tumor size, site of occurrence, malignancy or recurrence. Despite the relatively low expression rates of Pax-2 and Pax-8 in SFTs, their presence may contribute to an increased risk of misdiagnosis, especially in cases of DSFTs that exhibit a loss of the typical expression of CD34 and STAT6. Recently, it has also been reported in the literature that Pax-2 and Pax-8 exhibit a certain expression rate in other soft tissue tumors, such as synovial sarcoma (11), biphenotypic sinonasal sarcoma (12), *etc.* Therefore, the immunohistochemical positivity of Pax-2 and Pax-8 should be interpreted carefully in accordance with morphological assessment to ensure an accurate diagnosis. In addition, DSFTs can exhibit aberrant immunophenotypes due to their heterogeneous differentiation, such as the presence of desmin and myogenin in the embryonal rhabdomyosarcoma component (6). In the study by Mosquera and Fletcher (2), DSFT occasionally showed positive expression of CK and EMA in epithelioid differentiation area. Therefore, the DSFTs often present a diagnostic pitfall due to their unusual histological features, ambiguous immunophenotypes, and rarity. Consequently, further molecular testing is required.

Molecularly, DSFTs share the same pathogenic *NAB2-STAT6* fusions as classical SFTs. They have also been reported to exhibit multiple fusion variants, with at least 10 declared, the exon 6-exon 16/17 fusion being the most common. The fusion variant identified in this case, exon 5-exon 16, is relatively rare and has been documented in only a limited number of cases of SFTs (13). Consequently, the fact that multiple *NAB2-STAT6* fusion variants exist in DSFT suggests that the gene fusion variant is not a decisive factor responsible for the malignant behavior. Presumably, additional genetic damage is what leads to tumor dedifferentiation (2, 4). Akaike et al. (14) suggested that P53 overexpression plays a role in the acquisition of aggressive tumor phenotypes, and that *TP53* mutations are associated with the dedifferentiation process of SFT. Dagrada et al. (7)showed that aberrant P53 protein expression likely precedes the morphological malignant transformation, suggesting that *TP53* mutations may be a driver of dedifferentiated SFT. In addition, more than half of the DSFTs in the study by Yamada et al. (15) showed diffuse P16 and P53 protein expression, along with RB protein deletion. The authors suggested that the transformation of high-grade sarcomas is associated with genetic alterations in cell cycle regulatory proteins, such as P16 and Rb, and P53.

The morphologic spectrum of DSFT is more extensive and needs to be differentiated from a variety of malignant tumors. If the classical SFT region can be found, it can provide important clues for the diagnosis. However, the present case was a biopsy sample in which only dedifferentiated areas could be seen. So, the following diseases should be listed in the differentiated diagnosis. Firstly, the poorly differentiated carcinoma was taken into consideration owing to the epithiloid morphology, abnormal immunophenotype, and the anatomical relationship with kidney. However, the desmoplastic proliferation caused by cancerous infiltration was absent. Moreover, clinicians and radiologists ruled out the possibility of metastatic tumors and insisted that it was more inclined to be a soft tissue tumor. Combined with the clinical and imaging findings, the pathological diagnosis was reconsidered and the correct diagnosis arrived. Secondly, epithelioid malignant peripheral nerve sheath tumor (EMPNST) should also be considered as the differential diagnosis. Tumor cells of EMPNST are tightly arranged in the form of thick beams, nodules, or nests, with abundant eosinophilic cytoplasm, obvious nucleoli, and immunophenotypically diffusely positive for S-100 and SOX-10, with expression of CK-pan and retained positivity for H3K27me3. In molecular, 70% of the cases may have INI1/*SMARCB1* deletion. We excluded it according to the immunohistochemical findings. Additionally, the differential diagnosis of mesothelioma was also omitted. Mesothelioma usually diffusely affects the pleura and peritoneum, morphologically classified as epithelial, sarcomatoid, and biphasic subtypes. Immunohistochemically, tumor cells express a panel of mesothelial markers including Calretinin, CK5/6, WT1, and D2-40. Recent studies have indicated that the lack of BAP1 and MTAP expression, along with the homozygous deletion of *CDKN2A* identified through FISH, can effectively differentiate mesothelioma from benign proliferations. Nevertheless, these immunophenotypic and molecular characteristics were not observed in our auxiliary detection.

Regarding the essential auxiliary tests pertinent to our study, specifically the methodology of NGS, it is important to deliberate on the choice between DNA sequencing (DNAseq) and RNA sequencing (RNAseq). DNAseq identifies structural rearrangements at the genomic level, including intronic or intergenic breakpoints, making it indispensable for detecting fusions irrespective of transcriptional activity. Additionally, DNAseq is preferred when investigating fusions in archival samples with degraded RNA. However, DNAseq may yield false positives if the fusion is not functionally transcribed, and its sensitivity depends on sequencing depth, tumor purity, coverage gaps, and the assay’s ability to resolve complex structural variants. Whereas, RNAseq detects expressed fusion transcripts, confirming their functional relevance. It is highly specific for biologically active fusions and can detect some junctions missed by DNAseq. However, it may fail to detect fusions in samples with low tumor cellularity, low transcript abundance, or transcriptional silencing. Additionally, RNAseq cannot resolve mutations in non-transcribed regions or provide information about copy-number variations (CNVs). Consequently, the integration of DNAseq and RNAseq methodologies enhances both sensitivity and specificity, particularly in tumors characterized by known genomic complexity.

In terms of biological behavior, DSFT presented with a metastatic rate of about 34% (16) and a mortality rate ranging from 37.5% to 70%. However, whether its biological behavior is related to the dedifferentiated components or the percentage of dedifferentiated area still needs further study. In terms of treatment, complete surgical resection is the best treatment currently. Studies have shown that DSFT is resistant to antivascular therapy but has some responsive to chemotherapy (6, 17). In our case, the patient underwent the therapy of anlotinib, a targeted agent for anti-tumor angiogenesis and inhibition of tumor growth, but received non-significant effects. It is possible that certain novel therapeutic approaches (18), including immune checkpoint inhibitor therapies, have not yet been fully explored, and their potential significance warrants further investigation.

## Conclusions

Herein, we reported a retroperitonal DSFT with unexpected CK-pan and Pax-2 expression, mimicking high-grade clear cell renal cell carcinoma. This case highlights the diagnostic challenges posed by DSFTs, particularly when they occur in atypical locations and exhibit abnormal morphology and immunophenotypes. In such cases, comprehensive clinical, pathological, and imaging assessments, along with molecular testing, are essential for accurate diagnosis.

## Data Availability

The original contributions presented in the study are included in the article/supplementary material. Further inquiries can be directed to the corresponding authors.
